# “A dim recognition.” Religion as a font of psychological innovation

**DOI:** 10.1007/s12124-024-09858-4

**Published:** 2024-07-22

**Authors:** Lucas B. Mazur

**Affiliations:** 1 Jagiellonian University, Kraków, Poland; 2 Sigmund Freud University, Berlin, Germany

**Keywords:** Catholicism, Christianity, James Jackson Putnam, Religion, Scruples, Scientism

## Abstract

While religion constituted one of the main topics of interest for early social scientists, faith traditions have silently slipped from this central role. When religion now appears in psychological research, it is usually relegated to the position of either the object of psychological investigation (which psychology purports to “explain”) or a static piece in the empirical puzzle (as one variable among many when explaining clinical or social outcomes). In either case, religion is generally no longer seen as an equal partner to the social sciences in our attempts to better understand of the human condition. However, there are and have been voices within psychology that see this as unnecessarily myopic. James Jackson Putnam (1846–1918), an early supporter of the emerging field of psychoanalysis, advocated that psychology take seriously philosophy, metaphysics, and religion. Putnam’s objections to the narrowing of our view of human life in the spirit of scientism fell largely on deaf ears, and his call for psychology to include that which lies beyond the walls of empirical naturalism and reductionism remains relevant today. In as far as theoretical innovation in psychology is more of a creative recognition than true scientific discovery, philosophy and religion constitute tremendously rich, and unfortunately underappreciated, fonts of inspiration. Putnam saw in religion the “dim recognition” of “the creative spirit of the universe.” We briefly reflect on the example of obsessive–compulsive disorder and the much older religious concept of scruples, including approaches to mindfulness. This example is suggestive of the richness of psychological insights to be found in religion.

*The form which art, religion, and literature assume is determined by men's personal experiences and special cravings. The essential motive of art and religion is, however, the dim recognition by men of their relation to the creative spirit of the universe.* (Putnam, 1921, p. 298).

James Jackson Putnam (1846–1918) was a neurologist, founding member of the American Neurological Association (and its president in 1888), and founder of what would become the Department of Neurology at Harvard University. He was also an active supporter of note of the then newly emerging field of psychoanalysis and psychology in general. After the famous conference organized by G. Stanley Hall at Clark University in September 1909, attended by, among others, Sigmund Freud and Karl Jung, Putnam hosted Freud at his family vacation home in the Adirondack Mountains (Prochnik, 2006). In 1911, Putnam was elected the first president of the American Psychoanalytic Association. He was clearly a man of science, and he advocated for the field of medicine to take seriously the insights afforded by the newly emerging field of psychology (and psychoanalysis) (Putnam, 1915, 1921). He argued that psychological science was a path to new insights into human health and human nature more broadly. At the same time, Putnam argued that in pursuing this aim of better understanding humanity, psychologists should take seriously those aspects of human life which lie beyond the reach of direct empirical investigation, such as what we might broadly call philosophy, metaphysics, or religion (Putnam, 1915, 1921). He saw this second path as not one of *discovery*, which is the purview of science, but rather of *recognition*—and he poetically calls religion and art the “dim recognition” of the creative force of the universe. Despite his advocacy that psychology recognize the power of the philosophical, the spiritual, and the religious, he lamented how psychologists continued to ignore and even explicitly reject these fundamental aspects of human life (Putnam, 1915, 1921). After his passing, in an obituary for this “foremost advocate of psychotherapy” in the United States, Ernest Jones (the leading psychoanalyst in Britain at the time and the first English-speaking psychotherapist) wrote that “it was a genuine disappointment to [Putnam] that his views made so little impression on his psycho-analytical colleagues” (cited in Outler, 1954, p. 59). Putnam’s call for psychology to include that which lies beyond the walls of empirical naturalism remains relevant today.

Putnam’s enthusiasm for, and disappointment in, the development of psychology nicely illustrate the fresh, expansive, and inclusive promise of the field on the one hand, while on the other hand the self-imposed limitations that accompanied the field’s early and increasing embrace of scientism. This embrace of scientism implies allegiance to the myths woven into the scientific advances of the Enlightenment, which includes the (implicit) belief that the study of humanity really only began with the rise of psychology towards the end the nineteenth century (Kołakowski, 1989). Despite an increasing awareness of the limits of naturalism—especially as they perennially become so glaring as to be undeniable (e.g., within the “replication crisis”)—psychology nevertheless generally suggests that our psychological lives can be understood by taking them apart and rebuilding them, like an old radio. We have come to largely assume that psychological phenomena can be understood without a past and without an imagined, dynamic, and co-constructed future. We hear just such amnesia in the words of one of the founders of modern social psychology, George Herbert Mead:

The long and short of it is that the only reality of the past open to our reflective research is the implication of the present, that the only reason for research into the past is the present problem of understanding a problematic world, and the only test of the truth of what we have discovered is our ability to so state the past that we can continue the conduct whose inhibition has set the problem to us. (Mead, 1938, p. 97)

In the scientific spirit of the Enlightenment, innovation within psychology can at times come in the form of *discovery*, which is to say that psychological research can lead to truly new knowledge about human psychological processes. There are certainly areas of psychological research, such as that on memory, which have developed over the last several decades in a manner similar to what we more often see in medicine or the natural sciences. In such cases, we have come to know something truly new. However, most theoretical innovations in psychology tends to come in the form of *recognition*. This is to say that psychological innovation tends to involve shedding new light on what we in fact already know or knew. Sometimes the object of knowledge had been forgotten, having fallen into complete darkness, sometimes it had fallen only into shadow, and sometimes it was still in the light but simply out of our attention until the spotlight of psychology drew our gaze to it. There are large literatures on various “new” concepts in psychology, such as mindfulness or the subconscious, showing their long histories before their ostensible “discovery” within the field of psychology. Even the study of the psychological itself has a much longer history than the formalized field (Leahey, 2017).

To assert that psychological innovation tends to take the form of *recognition* is to acknowledge the value of what came before the emergence of the field of psychology and of what happened and continues to happen outside the walls of the academy and the clinic. It is to embrace history and to take an expansive, holistic view of human knowledge regarding human psychological processes. This is fundamentally different than the development of the natural sciences, engineering, or even medicine, where the novitiate is not required to learn about the history of the field. As a collective, psychologists also tend to forget the past, but in this case rather than encouraging discovery by channeling energies into the further development of new technologies or via the expansion of their application into new areas, etc., among psychologists this creates the illusion of discovery. Limited knowledge about philosophy and religion can make recognition, and even creative innovation, appear as if it were discovery.

In as far as psychological innovation is at heart a recognition, or at best what we might call a *re*discovery, to the extent that psychologists ignore areas outside the field, we are cutting ourselves off from fonts of potential inspiration. This includes such areas as music and art, as well as philosophy, and yes, even religion. Music and art have generally not been perceived as a threat to the scientific status of the social sciences, even if the scope and depth of their value for the study of the human condition has generally not been consistently appreciated or incorporated into psychology (Valsiner, 2023). However, the attempt by psychologists to distance the field from philosophy—a field they saw as a threat—is well known: “Wherever philosophy has led some field of research to maturity, the protege cut the leading strings. Such was the case at first with the natural sciences; then in modern times this differentiation increased” (Dilthey, 1954/1907, p. 12). In the early years of the social sciences, particularly psychology and sociology, philosophy was seen as the possessive parent who wanted their child to remain indoors, and the commitment of social scientists to empirical research (in the spirit of the natural sciences) was understood as the assertion of freedom from that stifling parental control. The increased specialization we see today within various subfields of psychology, along with the overall loss of well-rounded, classical education (the loss of *Bildung*), has also certainly further contributed to the perceived isolation—and thus assumed novelty—of contemporary psychology. This story is widely told, and yet arguably not currently appreciated enough, and certainly not lamented enough, within “mainstream” psychology (Valsiner, 2017). What is also generally underappreciated, however, is the aggression underlying the larger positivistic project psychology has attempted, and largely continues, to follow (Kołakowski, 2003). This found uniquely honest expression in Auguste Comte’s referral to metaphysics as “a shade of bastard theology” and a “kind of chronic transitional malady” (de Lubac, 1995, pp. 151–152). Such open and honest vitriol is rare, as the fundamental aggression of positivism tends to take on more subtle, implicit forms.

If by the words “nonsensical” or “meaningless” we wish to express no more, by definition, than “not belonging to empirical science,” then the characterization of metaphysics as meaningless nonsense would be trivial; for metaphysics has usually been defined as non-empirical. But of course, the positivists believe they can say much more about metaphysics than that some of its statements are non-empirical. The words “meaningless” or “nonsensical” convey, and are meant to convey, a derogatory evaluation; and there is no doubt that what the positivists really want to achieve is not so much a successful demarcation as the final overthrow and the annihilation of metaphysics. (Popper, 2002, pp. 12-13)

Compared with the more well-known tensions between philosophy and psychology, the rejection by psychology of metaphysics and religion, or what we might call the spiritual, is a story told even less often, and lamented even less, within mainstream psychology. Nevertheless, there is a strong case to be made that religion served not only as the soil from which psychology grew (as well as the natural sciences, Lindberg, 2008), but that it constitutes at least some of the main roots from which the field continues to live; that which provides sustenance and rootedness while remaining buried and thus unseen. For example, even in the work of George Herbert Mead, who very openly chose not to live the Christianity of his religious family, we arguably see positions carried over from that same faith tradition, such as his belief in the inherent goodness and unity of humanity—an implicit nod to the Christian roots of Western humanism. Regarding the work of this “non-religious” seminal social psychologist, we read the rather sarcastic comment that “it is certainly a high tribute to the strength of the Christian leaven that it could so raise the threshold of even Mead’s tough mind as to bring curiosity to its knees before the sacred alter of brotherhood” (Smith, 1932, p. 212). Whether or not one supports the claim that psychology inherently carries within itself—largely incognito—many a religious position (the struggle of which is nicely exemplified in Smith, 1932; regarding G. H. Mead in particular, see also Karier, 1984), what is particularly interesting is the degree to which the question of the relationship between psychology and religion has simply vanished from discussion. This is all the more interesting given the degree to which the work of early social scientists can be understood as either directly studying religion (e.g., William James) or as the photographic negative of religion which emerged from its rejection (e.g., Comte’s desire to literally replace religious holidays, monuments, and even the calendar with positivistic ones), or even from arguing its declining influence (e.g., á la Durkheim, Marx, Weber, or Freud for that matter). It is as though we have fully embraced Comte’s *Law of Three Stages* according to which humanity necessarily leaves behind religion and philosophy in order to embrace positivism—science writ large (very large). It is as though by embracing scientism, as a field we have quite literally succumbed to a form of amnesia wherein our memory retains only the faintest memories of philosophy and almost none of religion. We have rushed more and more towards the empirical, and become trapped in a sandstorm of data looking out at the world as if through a straw. Through this myopic view of humanity we see only singular psychological phenomena, one after another as if they were truly divorced from context and history, and we pride ourselves on what we take to be a more “objective” view of each grain of sand that flies before us. While various versions of interpretive social science, including cultural psychology, powerfully argue the limitations of naturalistic assumptions within the study of humanity (e.g., the search for causal laws, prediction, and replication), the reassertion of historicity found there rarely extends that one step beyond philosophy into religion. Thus, while from time to time we hear the voice of our parent, philosophy, through the howl of the empirical winds, we need listen all the more carefully to hear the distant shouts of our grandparent, theology. In such ways, we tend to drastically narrow the scope of the human condition. Susan Blow, whom Prochnik (2006) calls J. J. Putnam’s “philosophical mentor,” poetically expressed this sentiment as follows:

“I have often a rebellious feeling that my hopes and despairs and struggles and self-corrections and aspirations and moral imperatives are not accorded the respect given to pebbles and beetles, clouds and crystals. I AM AS MUCH A REALITY as any physical thing—and my loves, faiths, struggles, hopes are the supreme reality of me.” (Susan Blow, cited in Prochnik, 2006)

An openness to philosophy and faith traditions is one way to encourage psychology to embrace the broader reality of which Blow writes. Von Fircks (2023) provides an example of how philosophical, metaphysical, and religious traditions can be suggestive of theoretical innovation within psychology and can even be a partner therein. More particularly, he explores the possibility of combining the notion of (*wu wei*) mindfulness with George Herbert Mead’s (“scientific”) social psychological understanding of the self, and he includes reflections on his own personal experiences to a degree rarely given voice today in psychological writing. This honest, personal engagement with both psychology and religion attests to a holistic, inclusive approach to innovation within our study, not just of psychology, but of the human condition.

While such concepts as *mindfulness* suggest the freshness of discovery, they also reminding us of both the collective past—of “ancient wisdom”—and of that which each of us already inherently knowns or can come to know over the course of life. While it can take the form of a practice to be taught and learned, it is also something that inherently emerges from life itself. If one is open and listens carefully, we can hear echoes of similar or related phenomena from within various religious traditions. In the quotation below we read of one man’s employment of what we might call “mindfulness” in his struggle with the religious concept of *scruples*, that is, obsessive thoughts on, and worry about, one’s own ostensible sins:

“Why did you expose yourself and others to those evil thoughts? And if you did so, too bad. Do not do that again. Go, confess and your peace will return.” However, I did not respond to those suggestions. My saboteur carried on a monologue, trying to turn that monologue into a dialogue. I heard him well, but I did not answer him, I did not enter a dialogue. From time to time, I called on God’s help. Apart from the method of not establishing contact, I made use of yet another technique: I avoided using violence again my psyche, I did not dismiss these penetrating thoughts, I took a passive position; namely, I allowed them to wash over me like a wave, while simultaneously not giving up within my will. In short—I endured in silence. (Działa, 1982, p. 39-40)

Here we see Christian approaches to silence, suffering, sin, and salvation, enacted to create a form of proactive but passive inertia in the face of what would otherwise be distracting and even harmful thoughts. Thus, these theological particulars created a form of mindfulness that allowed this man to pass through a difficult period of obsessive thinking. There is a considerable literature on the similarities and differences between the mindfulness, meditation, and contemplative practices found in Catholicism and various Eastern religious traditions, perhaps most famously in the work of Thomas Merton (Keenan, 2017). The example of mindfulness provided in the quotation above is arguably in some ways different in its theological and psychological foundations from what we see in the use of the Taoist concept of *wu wei* mindfulness as explored by von Fircks (2023). For example, while in Christianity both acts and thoughts can be sinful, in the case of scruples, obsessive thoughts can be understood as coming from the “outside”—both in a psychological and theological sense. This can be understood in a psychological sense in several ways, such as in the idea that the obsessions have a biological foundation or that they are symptomatic of a mental disorder, and thus that they are something apart from the psychological self. In a theological sense they can be understood as challenges to more fundamental aspects of one’s Christian faith, such as trust in God and faith in the redeeming power of God’s love (e.g., via the sacrament of confessions/reconciliation), and thus they can be seen as a challenge to the moral self, rather than an inherent part of it. In other words, while one remains morally responsible for one’s own actions and even one’s own thoughts, the moral evaluation of scruples can shift from an assessment of those thoughts themselves (or about the ostensible sins suggested in those thoughts) to an evaluation of one’s struggle with those problematic thoughts themselves. Rather than struggling to respond to what are ultimately non-existent sins (as suggested again and again by the obsessive thoughts), the scrupulant is challenged to assert his or her commitment to the moral order of God (which implies that the scruples are at their core not a part thereof and that the sins to which they give testament, whether real or not, are of a lesser order).

While obsessive–compulsive disorder has in effect generally become the umbrella term used by psychologists for various obsessive–compulsive disorders, including scrupulosity, there is no consensus on the exact nature of that relationship (Greenberg & Huppert, 2010; Miller & Hedges, 2008). What is particularly interesting is that scrupulosity has a particularly long history: “Ironically, scrupulosity has perhaps one of the longest and richest histories of any psychological disorder” (Miller & Hedges, 2008, p. 1042; see also Ciarrocchi, 1995; Greenberg & Huppert, 2010; Santa, 1999). There are a handful of well-known accounts of what we would today call scruples as experienced by famous historical figures (e.g., St. Ignatius of Loyola, St. Thérèse of Lisieux, John Bunyan, Robert Boyle). What is more, there is a significant Catholic literature on scruples going back centuries (Ciarrocchi, 1995; de Lehen, 1888; Duguet, 1718; Santa, 1999). The wealth of insights within the Catholic tradition regarding scruples, and thus potentially regarding obsessive–compulsive disorders more broadly, is further suggested by the following rather simple points, a short list which is by no means exhaustive:

1. Catholicism has a centuries-old history of reflecting on the kinds of obsessive thoughts found in scruples. The study of such matters did not begin in the 19^th^ century with rise of modern psychology.

2. Within the large Catholic literature on these matters, numerous descriptions of such obsessions have been recorded, which would likely make for interesting and inspiring analysis.

3. Numerous ways of addressing such obsessions have been developed within Catholicism, which could very well be useful for theoretical innovation within psychological.

4. The Catholic priesthood, especially priests particularly knowledgeable of scruples, constitutes a living and breathing body of knowledge and practice that could very well help to better inform our psychological understanding of such obsessions and compulsions and the mindfulness practices that can be employed to address them.

While religious people, including members of religious orders (e.g., priests, brothers, nuns), can explicitly incorporate psychology into their lives and even into their spiritual, religious work, it is rare that psychologists so overtly incorporate religion into theirs. In other words, while psychology can easily enter religious discourse (e.g., into the religious conversations of religious people, and even within explicitly religious contexts such as churches), it is extremely rare that religion enters as seamlessly into the discourse of psychology (e.g., during psychology conferences, research projects, or publications). The point here is that religion constitutes a truly rich trove to explore, one that remains severely understudied and underappreciated by (secular) psychologists. Importantly, just as psychological concepts can be misunderstood, distorted, and misused when taken out of the hands of psychologists, religious concepts can similarly by misconceived and otherwise abused. For example, by taking various mindfulness traditions out of their original philosophical and/or religious context, and then using them to achieve concrete goals (e.g., in the spirit of clinical or even popular psychology), we run the risk of in effect radically changing the very nature of those traditions. While not a problem in itself, this can make them in effect too easily “digestible” to secular psychologists today, and devoid of the innovative nourishment they might otherwise provide in their difference. Thus, as has become a mantra in some fields (most classically anthropology), such cross-fertilization requires truly learning about these concepts from the “inside.” Catholicism’s approach to scruples cannot be understood apart from its wider context and history, including its theology. For example, scruples can only really be understood within the context of such concepts as sin, repentance, forgiveness, grace, sacrifice, and salvation. Just as priests who would benefit from a knowledge of psychology cannot simply make use of psychological terms ripped out of their larger theoretical contexts, psychologists who would benefit from the insights of religion should acquire a deep, insider knowledge of those faith traditions. This is not to say that psychologists, either as psychologists or simply as people, need be religious, but simply that by so thoroughly ignoring religion we are in effect ignoring both an important part of history, even our own history as a field, and an important partner that we can not only teach, but from whom we ourselves stand to learn a great deal.

While a richer exploration of scruples, and mindfulness practices related thereto, is unfortunately beyond the scope of this brief piece, what is particularly interesting about the case of scruples is that there exists a ready-made body of (religious) literature and a body of specialists (i.e., the priesthood, clergy, rabbis, imams) that might in effect guide psychologists into the topic from within these religious traditions. These sources are a potential treasure-trove of information regarding scruples and how to address them, such as various mindfulness practices. What is more, these approaches to scruples may prove inspiring of psychological innovation more broadly.

## Conclusion

Today, with few exceptions, religion is generally rejected out of hand by psychologists as being either irrelevant for psychology or, at best, as being either the object of psychological investigation (as seen in the “psychology of religion”) or a static piece in the empirical puzzle (e.g., as one variable among many when explaining clinical or social outcomes). Religion, like philosophy, is generally not seen as a partner from whom we can learn or a forefather to which we are indebted. What is particularly striking about this is the levity with which such dismissal now happens, that is, how little thought is given to this position. However, this silence is of considerable consequence. We continue to cling, and arguably we even cling more fiercely than ever, to the certainty science seems to promise when applied to the social “sciences.” In 1910, Wilhelm Dilthey (2002/1910, p. 347) poetically described this tendency to cling to certainty as follows: “In the midst of changing seasons and the storms of change around us, we seek solid walls to protect us, even if they set limits on us.” In our search for theoretical insights in psychology, the trick is to make the cracks in these walls of presumed certainty wide enough that we might make out what lies beyond—that we might be afforded a glance upon, and the dim recognition of, our relationship with the creative spirit of the universe.

## Data Availability

No datasets were generated or analysed during the current study.
